# Endometriosis-Related Infertility: The Role of the Assisted Reproductive Technologies

**DOI:** 10.1155/2015/482959

**Published:** 2015-07-09

**Authors:** Eric S. Surrey

**Affiliations:** Colorado Center for Reproductive Medicine, 10290 Ridge Gate Circle, Lone Tree, CO 80124, USA

## Abstract

The assisted reproductive technologies, particularly in vitro fertilization (IVF), represent the most efficient and successful means of overcoming infertility associated with endometriosis. Although older studies suggest that IVF outcomes are compromised in endometriosis patients, more contemporary reports show no differences compared to controls. The exception may be evidence of poorer outcomes and diminished ovarian response in women with advanced disease, particularly those with significant ovarian involvement or prior ovarian surgery. Prolonged pre-IVF cycle suppressive medical therapy, particularly gonadotropin releasing hormone agonists, appears to improve success rates in a subset of endometriosis patients. However, as of yet, there is no diagnostic marker to specifically identify those who would most benefit from this approach. Pre-IVF cycle surgical resection of nonovarian disease has not been consistently shown to improve outcomes with the possible exception of resection of deeply invasive disease, although the data is limited. Precycle resection of ovarian endometriomas does not have benefit and should only be performed for gynecologic indications. Indeed, there is a large body of evidence to suggest that this procedure may have a deleterious impact on ovarian reserve and response. A dearth of appropriately designed trials makes development of definitive treatment paradigms challenging.

## 1. Introduction

The impact of endometriosis on fertility and proposed mechanisms of this phenomenon have been addressed elsewhere in this paper. The assisted reproductive technologies and, more specifically, in vitro fertilization (IVF) represent the most successful means of achieving conception in endometriosis patients struggling with infertility. This approach bypasses anatomic distortion, potential compromise in tubal function, and aberrations in the peritoneal environment associated with this disease. In this paper, we shall explore the impact of endometriosis on IVF cycle outcomes as well as whether surgical or medical management of endometriosis per se can impact success rates.

## 2. The Impact of Endometriosis on IVF Outcome

The issue of whether the diagnosis of endometriosis has a negative impact on the outcome of IVF has not been resolved. Although several early studies suggested poorer outcomes in comparison to controls, other showed no significant differences [1]. A meta-analysis performed by Barnhart et al., which included only clinical trials published from 1983–98, calculated that the number of oocytes obtained as well as fertilization, implantation, and pregnancy rates was lower after IVF in patients with endometriosis than in controls with tubal factor infertility [2]. It is important to note that pregnancy rates in both groups were extremely low (12.7% versus 18.1%) and do not reflect the significantly improved outcomes which are typically achieved in current practice. A more contemporary Norwegian retrospective analysis from a single center reported virtually identical live birth rates after IVF for patients with endometriosis versus tubal infertility (66.0% versus 66.7%) [3]. Implantation rates were also similar between the groups. Barcelos et al. more recently noted no differences in the percentage of meiotic abnormalities in in vitro matured oocytes from endometriosis or control patients after ovarian stimulation [4].

The 2012 Clinic Summary Report of the Society for Assisted Reproductive Technology reflects no real differences in implantation or pregnancy rates when comparing the subgroup of patients with endometriosis to the aggregate of patients with all diagnoses undergoing IVF in the United States [5] (Table 1). It is important to note that the database does not reflect disease stage, past therapy, or presence of ovarian endometriomas. Also of note is the fact that this summary reports that only 3% of the cycles performed in 2012 in the United States were associated with a primary diagnosis of endometriosis which is clearly an underestimate. This discrepancy can be attributed to the decreasing role of diagnostic laparoscopy as part of the infertility evaluation leading to these patients being classified with a diagnosis of either “unexplained infertility” or under some other primary diagnosis that may have been considered to have a greater impact on fertility.

One of the fundamental flaws of these reports is the failure to uniformly control for other infertility variables that could affect outcome including ovarian reserve and sperm function testing, uterine evaluation, untreated hydrosalpinges, and ovulatory factors. In addition, adenomyosis is frequently found in patients with endometriosis and its presence may have a deleterious impact on implantation [6, 7].

The summary data described above does not address the question of whether patients with more severe disease may have different outcomes than those with less extensive endometriosis. Barnhart et al. in the previously described meta-analysis of clinical trials from 1983–98 compared outcomes in patients with American Society for Reproductive Medicine (ASRM) stages I-II to those with stages III-IV endometriosis [2]. Implantation and pregnancy rates as well as number of oocytes retrieved were significantly lower in the latter group. The results from earlier trials that addressed the impact of more severe endometriosis were confounded by the use of laparoscopic retrieval techniques which, in the face of distorted anatomy and dense pelvic adhesions, may have limited the ability to adequately retrieve oocytes in patients with more extensive disease [1]. Nevertheless, these outcomes were confirmed in a more recent trial by Kuivasaari and colleagues who reported significantly lower implantation rates in patients with ASRM stages III-IV endometriosis versus controls with ASRM stages I-II endometriosis or tubal factor infertility [8].

Opøien et al. noted in a large retrospective trial that in comparison to tubal factor controls, patients with ASRM stages I-II disease had lower fertilization rates whereas patients with more severe disease had fewer oocytes retrieved despite requiring higher gonadotropin doses [9]. Nevertheless, pregnancy and live birth rates were not different. A contemporary meta-analysis of 27 observational studies reported a reduction in fertilization rates only (RR = 0.93, 95% CI: 0.87–0.99, *P* = 0.03) in women with stage I/II endometriosis undergoing IVF [10] In this same report, patients with stage III/IV endometriosis were noted to have a decrease in implantation rates (RR = 0.79, 95% CI: 0.67–0.93, *P* = 0.006) and clinical pregnancy rates (RR = 0.79, 95% CI: 0.69–0.91, *P* = 0.0008). This analysis did not evaluate live birth rates and is weakened by the heterogeneity of comparison groups which were defined as “women without endometriosis.” The poorer response in these patients may reflect aberrations in granulosa cell estrogen and progesterone receptors that has been reported in women with more extensive disease [11].

The presence of ovarian endometrioma(s) may represent a confounding variable in assessing IVF outcome. A decrease in ovarian response in patients with endometriomas necessitating higher gonadotropin doses has been described [12, 13]. It has been suggested that this impact was exacerbated by the size and number of lesions. However, the impact of these lesions cannot be addressed independently in that it is extremely rare for a patient to only have an endometrioma as the sole manifestation of endometriosis in the absence of peritoneal disease. Studies which rely solely on ultrasound diagnosis can neither make a definitive diagnosis of the presence of an endometrioma nor can they rule out the presence of additional disease, the presence of which can only be assessed surgically.

Benaglia and coworkers also reported that although responsiveness to gonadotropin stimulation and number of oocytes retrieved were reduced in women with bilateral endometriomas in comparison to controls without evidence of endometriosis or endometriomas, the rates of top quality embryos, implantation, clinical pregnancy, and live birth did not differ between the groups [14]. This finding has been confirmed by others [15, 16]. There is a dearth of evidence to suggest an incidence of other differences in response among endometriosis patients such as premature progesterone rise or LH surge.

Filippi and colleagues assessed developmental competence of oocytes obtained from ovaries with unilateral unoperated endometriomas in comparison to those obtained from the unaffected contralateral ovary [17]. No differences in number of oocytes obtained, fertilization rates, or resulting viable or high quality embryos were noted. In a classic large retrospective trial, Olivennes et al. reported that the presence of an endometrioma had no impact on any cycle outcome parameters in comparison to tubal factor controls [18]. In contrast, a more recent large study of 2245 patients noted that, although stages III-IV patients in general fared as well as controls, those with endometriosis not only required higher gonadotropin stimulation doses but exhibited a trend towards lower pregnancy and live birth rates [9].

Another variable which has only recently been addressed is that of deeply infiltrative endometriosis (DIE), a parameter that is not included in the purely visual ASRM staging system. Ballester et al. noted that the presence of DIE resulted in significantly lower IVF pregnancy rates than in patients with only superficial disease (58% versus 83%, *P* = 0.03) [19, 20]. However, in this trial, neither the presence, size, nor laterality of endometriomas had any impact on outcome. These investigators suggest that the presence of DIE was the strongest predictor of IVF outcome (odds ratio [OR] 0.26, 95% CI: 0.07–0.9, *P* = 0.006).

Difficulties in comparing the results of these trials include the inherent weakness of the ASRM scoring system which does not specifically address extraperitoneal or deeply infiltrating disease, variability in endometrioma size, number, and laterality, and the use of ultrasound versus surgical diagnosis of endometriomas. These confounding variables may play differing impacts on outcomes but, in general, have not been consistently addressed.

## 3. The Impact of Gonadotropin Stimulation on Endometriosis

Given the well-accepted relationship between estrogen stimulation and the maintenance as well as progression of endometriosis, one could question whether the highly elevated estradiol levels induced by gonadotropin stimulation could exacerbate underlying disease. The data which address this issue are limited but encouraging.

One study noted that 3–6 months after completion of an IVF cycle, overall endometriosis symptom scores were unchanged with 11% of patients reporting worsening and 77% reporting improvement [21]. Endometrioma size also remained stable. D'Hooghe and coworkers performed a life table analysis of patients with stage III/IV endometriosis who underwent gonadotropin stimulation and reported that, despite using higher gonadotropin doses resulting in higher mean circulating estradiol levels, cumulative disease recurrence was lower in IVF than in intrauterine insemination (IUI) cycles [22].

## 4. Impact of Medical Therapy for Endometriosis on IVF Outcome

A host of medical interventions has been demonstrated to have benefit in alleviating, if not eliminating, symptoms associated with endometriosis. As has been addressed elsewhere in this text, such agents as danazol, gonadotropin-releasing hormone agonists (GnRHa), and progestins have not been shown to enhance pregnancy rates associated with natural or stimulated cycles in infertile women with endometriosis who are not undergoing IVF. This paradox could be explained by one of two mechanisms. Either the etiology of endometriosis-related infertility is not suppressed by traditional medical interventions or the negative impact of endometriosis on fertility returns with resumption of ovulation after medications are discontinued. If the latter were the case, then medical suppression followed immediately by in vitro fertilization should overcome the problem. A variety of studies have shown that this may indeed be the case.

The largest body of work has addressed the prolonged use of GnRHa prior to initiation of gonadotropin stimulation for the assisted reproductive technologies. In a prospective randomized multicenter trial, Surrey et al. evaluated 41 patients with surgically confirmed endometriosis [23]. Twenty-five were treated with a three-month course of a GnRHa prior to ovarian stimulation and IVF. Twenty-six underwent standard ovarian stimulation prior to IVF. Despite having a higher percentage of patients with more advanced disease, the group administered a prolonged course of GnRHa exhibited a trend towards higher implantation rates (42.7% versus 30.4%) and significantly higher clinical pregnancy rates (80% versus 53.9%, *P* < 0.05) than controls.

Similar outcomes have been reported by others [24–29]. Three of the prospective randomized trials including 163 patients were assessed in a meta-analysis performed by Sallam et al. [30]. Prolonged use of GnRHa resulted in enhanced clinical pregnancy (OR 4.28; 95% CI: 7.0–9.15) and live birth (OR 4.28; 95% CI: 1.08 ± 8.22) rates.

A more recent retrospective analysis from the Netherlands compared 68 patients treated with at least 3 months of prolonged GnRHa therapy to 45 controls [31]. They reported a benefit (which did not reach clinical significance) only when fresh and cryopreserved embryo transfers were combined. In a prospective randomized trial, Rickes and coworkers assessed the role of prolonged GnRHa therapy for 6 months prior to either IVF or IUI after surgical treatment of endometriosis [29]. A statistically significant benefit was noted only among patients with more severe disease (stages III and IV) who subsequently underwent IVF.

Comparing outcomes among these trials is extremely difficult. Study designs and inclusion criteria vary. There are significant variations in the duration of GnRHa therapy amongst these trials which were published over a 24-year period during which clinical and laboratory practices as well as overall outcomes from the assisted technologies have significantly changed (Table 2).

The mechanism of action by which administration of prolonged GnRHa could impact IVF outcome has not been definitively demonstrated. Previous studies have shown that GnRHa may have an impact on suppressing peritoneal fluid inflammatory proteins, metalloproteinase inhibitor concentrations, and increasing proapoptotic protein expression [32–34]. Endometrial effects have also been postulated. Wang et al. reported that GnRHa significantly decreased nitric oxide synthesis expression within the endometrium [35]. Lessey had reported that women with endometriosis were more likely to have aberrant endometrial expression of *β*_3_ integrin and that a 3-month course of GnRHa allowed for a 64% rate of returned expression [36]. These results have been confirmed in a murine model [37]. Farrell et al. demonstrated that an 8-week course of GnRHa and norethindrone acetate resulted in 9 ongoing IVF pregnancies in 11 patients with absent endometrial *β*_3_ integrin expression [38]. We had previously demonstrated a 48.6% prevalence of aberrant expression in a group of consecutive high risk IVF patients with endometriosis and/or prior failed embryo transfer despite good embryo quality [39].

In order to assess the predictive value of endometrial *β*_3_ integrin expression in determining which endometriosis patients might benefit from precycle prolonged GnRHa therapy, Surrey and colleagues randomized endometriosis patients either to a 3-month course of GnRHa or to proceeding directly to ovarian stimulation after obtaining endometrial biopsies for *β*_3_ integrin [40]. Unfortunately, this study demonstrated that the biopsy results were of little value in predicting which patients would benefit from GnRHa therapy. One confounding variable in the study design was that patients in the control group underwent immediate gonadotropin stimulation after endometrial biopsy. Others have suggested that the biopsy itself may have a beneficial impact on enhancing implantation in patients with prior implantation failure [41]. A more appropriate design, which would potentially have had a negative effect on patient recruitment, would have been to have the control group also wait for three months prior to initiating gonadotropin stimulation in order to mitigate any impact of the biopsy per se.

Other interventions have also been employed in patients with abnormal integrin expression. Tei and coworkers administered danazol 400 mg daily for 12 weeks to 9 patients with aberrant expression and repeated IVF failures [42]. A significant increase in integrin expression in the first posttreatment ovulatory cycle was noted although pregnancy rates were not reported. A more recent retrospective trial employed a brief course of an aromatase inhibitor during the beginning of gonadotropin stimulation to integrin expression negative patients undergoing IVF and reported similar clinical pregnancy and live birth rates as those who were integrin positive [43].

The use of oral contraceptives as pretreatment has also been reported. de Ziegler et al. noted higher pregnancy rates after a 6–8-week pre-IVF cycle course of oral contraceptives in patients with either surgically diagnosed or sonographically suspected endometriosis than in controls without endometriosis (35% versus 17.9%, *P* = 0.01) [44]. The lack of confirmed diagnosis of endometriosis and retrospective design does represent confounding variables in this trial.

A recent publication has suggested that other markers such as mid-secretory endometrial leukemia inhibitor factor may be strongly associated with women who exhibit compromised integrin expression and might also be used in combination to better diagnose those patients with endometrial abnormalities that could potentially benefit from intervention [45].

There are several difficulties in interpreting the aforementioned trials. There have been no comparative studies among agents. The optimal duration of therapy has not been established by comparative trials. The ideal subset of endometriosis patients who would benefit from medical intervention has not been ascertained although it would appear that those with more severe disease and/or with prior evidence of implantation failure might be the best candidates.

## 5. Impact of Surgical Management of Endometriosis on IVF Outcome

The effect of surgical management on endometriosis associated infertility has been addressed elsewhere in this issue. The impact of this approach on IVF outcomes has not been evaluated extensively. It would be appropriate to separate outcomes from surgery associated with and without endometrioma resection. We shall first address the latter.

The logic behind surgical resection of peritoneal disease would be to minimize any deleterious effects that peritoneal implants or their secretory products might have on oocyte quality, embryo development, or implantation. Unfortunately, the evidence to support the fact that any of these phenomena actually occur is lacking.

Most studies on surgical management are retrospective in nature. Comparisons between the outcomes of various investigations are limited by variations in surgical techniques (i.e., ablation versus resection), completeness of removal of the disease, and differences in IVF laboratories. We had previously reported that IVF implantation rates were not affected by the time interval from surgical resection of endometriosis in the absence of endometriomas to oocyte aspiration (up to 5 years) or by endometriosis scores [46]. Implantation and ongoing pregnancy rates were similar between a group of patients who had undergone resection within 6 months of oocyte aspiration and a second group who had undergone resection greater than 6 months to 5 years before oocyte aspiration (implantation rates 34.6% versus 36.7%). This finding has been confirmed by others [47].

Contrasting reports have shown that precycle surgical intervention may be beneficial. In a retrospective trial, Opøien and coworkers evaluated outcomes in a single center from patients with stage I/II endometriosis who underwent surgical resection or controls who underwent diagnostic laparoscopy only before IVF/ICSI [48]. Significantly higher clinical pregnancy (40.1% versus 29.4%, *P* = 0.004) and implantation (30.9% versus 23.9%; *P* = 0.02) rates were achieved in those who underwent resection. Another investigative team, evaluating 825 patients with endometriosis-related infertility over a seven-year period, reported that overall pregnancy rates were significantly higher in patients who underwent surgical resection and then IVF in comparison to those who underwent surgery alone, IVF alone, or no treatment (65.8%, 54.2%, 37.2%, and 11.8%) [49]. It is a bit surprising that pregnancy rates from surgery alone would be so much higher than with IVF alone. However, it is important to note that the pregnancy rates reported were not per cycle but were cumulative and the mean time to achieve pregnancy after surgery was 11.8 ± 12.1 months (range 1–66 months).

Patients with more deeply invasive endometriosis may represent a separate subset. Bianchi et al. reported on a cohort of patients who underwent extensive resection of DIE prior to IVF [50]. Implantation and pregnancy rates were significantly higher in patients who underwent resection but fewer oocytes were retrieved and higher gonadotropin doses were required in that group. One problem with this trial is the lack of surgical confirmation of disease in the control group. Although not specifically limited to IVF, Douay-Hauser et al. reported that extensive surgery for DIE had no effect on global fertility but did result in a higher rate of complications than in those who had undergone less extensive procedures [51].

The lack of randomized trials regarding pre-IVF cycle surgical management of endometriosis makes it difficult to recommend this approach unless symptom relief is the primary goal.

One circumstance in which there is little controversy regarding surgical intervention is the presence of distal tubal occlusion with hydrosalpinx which can be secondary to endometriosis. A recent Cochrane meta-analysis of randomized controlled trials has concluded that laparoscopic salpingectomy or proximal tubal occlusion in women with hydrosalpinges results in IVF pregnancy rates which are similar to those in women without hydrosalpinges and significantly greater than when the hydrosalpinx is left untreated [52]. Outcomes have been shown to be similar after proximal occlusion or salpingectomy [53]. Case series have also reported success after hysteroscopic tubal occlusion with placement of microinserts, although this is an off-label use of the device [54].

The surgical management of the endometrioma and specifically its impact on IVF outcome is fraught with controversy. There are investigators who have suggested that these lesions may represent a different pathophysiologic process than other manifestations of endometriosis [55, 56]. Arguments that have been made to support precycle endometrioma resection include (1) inability to access follicles at oocyte retrieval, (2) concern that inadvertent exposure of oocytes to endometrioma fluid could have a deleterious impact on oocytes, and (3) the view that endometrioma resection would improve IVF outcome. The first case may be true in the face of large lesions (i.e., greater than 4-5 cm in mean diameter). With regards to the second situation, at least one investigative team has shown that exposure of oocytes to endometrioma fluid has no impact on rates of fertilization on early embryo development [57].

With regards to the third rationale, two meta-analyses have been performed to assess the impact of endometrioma resection on IVF outcomes. Tsoumpou and coworkers analyzed five studies which compared surgical resection of endometrioma to no treatment and demonstrated no significant differences in response to gonadotropin stimulation or in clinical pregnancy rates [58]. Benschop et al. performed a Cochrane meta-analysis involving 312 patients in four eligible studies and confirmed that surgical management of endometriomas resulted in no benefits for a subsequent IVF cycle [59]. It is important to note that these trials did not control for the potentially confounding variables of specific surgical techniques (aspiration, stripping and total excision, partial resection, and ablation), endometrioma size, or laterality. Indeed, it has been stated that the only indication for removing an endometrioma greater than 3 cm in mean diameter before IVF would be to treat painful symptoms or to improve ovarian access [60]. Garcia-Velasco and Somigliana proposed a series of well-considered indications for surgical intervention [61] as listed below. 

*Proposed Indications for Resection of a Suspected Endometrioma prior to IVF (Modified from [61]):*

- rapid growth,
- suspicious features noted on ultrasound,
- painful symptoms that can be attributed to the mass,
- potential for rupture in pregnancy,
- inability to access follicles in normal ovarian tissue.

Needless to say, if endometrioma resection is performed, it is critical to proceed conservatively and to minimize compromise of ovarian blood supply and preserve normal ovarian tissue [62].

Not only has excision of endometriomas failed to have been shown to be of benefit, but there is compelling evidence to suggest that such surgery may exert a deleterious effect. The majority of evidence is based on excision of lesions at least 3 cm in diameter [63]. However, the rationale for resecting smaller stable lesions without suspicious characteristics can be called into question. Nevertheless, Somigliana and colleagues reported a 53% reduction in response to gonadotropins in ovaries which had been operated upon regardless of size of the cyst with an absence in follicular development in 13% of cases after excision of unilateral endometriomas [64, 65]. Although these outcomes have not been demonstrated by all, two literature analyses are telling. In one review, nine of 11 studies showed a statistically significant postoperative decline in serum anti-Müllerian hormone (AMH) levels, which was exacerbated by excision of bilateral lesions [65]. In a more recent meta-analysis, Muzii et al. extracted data on 597 patients from 13 of 24 evaluated studies [66]. Despite a high degree of heterogeneity amongst the studies, they noted that the antral follicle count was inherently lower in the affected ovary. This difference only reached statistical significance after surgery.

Thus, clinicians should carefully consider the risks and benefits of pre-IVF cycle endometrioma resection given the lack of compelling data to support this procedure beyond the aforementioned circumstances. Patients should be thoroughly counseled regarding risks to ovarian reserve and response particularly in those who already have evidence of compromise.

## 6. Conclusions

In general, IVF represents the most successful, but not only, approach to overcome endometriosis-related infertility. Contemporary evidence would suggest that women with this disorder have similar cycle outcomes to other patients going through IVF. However, patients with extensive ovarian disease and those who have undergone multiple prior ovarian surgeries are more likely to have diminished ovarian reserve and response to gonadotropins. It is therefore critical for clinicians to perform a thorough assessment of ovarian reserve, tubal patency, sperm function, and the uterine cavity prior to initiating therapy.

There is good evidence to suggest that prolonged administration of GnRHa to at least a subset of patients with endometriosis may improve cycle outcome. Unfortunately, given the added expense and delay associated with this approach, it would be ideal to identify the appropriate patient subset and duration of therapy. In the absence of adequate data, it would be logical to consider this approach in endometriosis patients with prior failed cycles as well as those who are symptomatic and with more severe disease. Other agents such as danazol, aromatase inhibitors, and oral contraceptives have been less extensively evaluated and, therefore, their use cannot be recommended at this time.

Precycle surgical ablation or resection of asymptomatic disease does not appear to be generally beneficial aside from achieving symptom relief, although heterogeneity amongst studies makes data analysis challenging. An exception to this may be the resection of deeply infiltrative endometriosis, although the number of studies is small.

Endometriomas should not be resected to enhance IVF outcome and much evidence suggests a deleterious effect of surgery on ovarian reserve and response. The indications for this procedure should be limited to suspicious appearance, rapid growth, progressive symptoms, and an inability to aspirate follicles due to the size of the lesion. Conservative surgical approaches taking great care to avoid compromise of normal ovarian tissue and blood supply are critical.

The need for additional well designed prospective randomized trials reflecting contemporary IVF laboratory practices is critical to allow clinicians to better care for these challenging patients.

## Figures and Tables

**Table 1:** Endometriosis and IVF: fresh embryo transfer with nondonor oocytes 2012 SART Registry^*^.

	Age: <35	35–37	38–40	41-42
Implantation rate (%)				
Endometriosis	36.2	26.6	15.8	9.0
All diagnoses	37.5	27.0	18.4	9.8
Live birth rate (%)				
Endometriosis	41.8	32.6	20.5	10.0
All diagnoses	40.7	31.3	22.2	11.8

^*∗*^Modified from 2012 SART Clinical Summary Report [5].

**Table 2:** Impact of prolonged GnRHa prior to IVF in endometriosis patients.

1st author (reference)	Year	GnRHa duration	Patients/cycles	Clinical pregnancy (%)			Design
				No GnRHa	Luteal GnRHa	Prolonged GnRHa	
Remorgida [24]	1990	6 months	60/60	33	32	56	Prospective randomized
Dicker [25]	1990	6 months	64/64	5^*^	—	33	Prospective randomized
Nakamura [26]	1992	126 ± 57 days	32/32	—	27^*^	67	Retrospective
Marcus [27]	1994	2–7 months	84/181	—	11	35	“Semirandomized”
Chedid [28]	1995	3 months	145/171	23^*^	39	46	Retrospective
Surrey [23]	2002	3 months	51/51	—	53.8^*^	80	Prospective randomized
Rickes [29]	2002	6 months	47/82	—	47	75	Postoperative
			Stage I/II		50	56	Prospective randomized
			Stage III/IV		40^*^	82	
Van der Houwen [31]	2014	3–6 months	113/113	Fresh	22.2	25	Retrospective
			Fresh + cryopreserved		22.2	35.3	

^*∗*^ *P* < 0.05 versus prolonged GnRHa.

## References

[1] Surrey E. S. (2013). Endometriosis and assisted reproductive technologies: maximizing outcomes. *Seminars in Reproductive Medicine*.

[2] Barnhart K., Dunsmoor-Su R., Coutifaris C. (2002). Effect of endometriosis on in vitro fertilization. *Fertility and Sterility*.

[3] Omland A. K., Åbyholm T., Fedorcsák P. (2005). Pregnancy outcome after IVF and ICSI in unexplained, endometriosis-associated and tubal factor infertility. *Human Reproduction*.

[4] Barcelos I. D., Vieira R. C., Ferreira E. M., Martins W. P., Ferriani R. A., Navarro P. A. (2009). Comparative analysis of the spindle and chromosome configurations of in vitro-matured oocytes from patients with endometriosis and from control subjects: a pilot study. *Fertility and Sterility*.

[5] Society for Assisted Reproductive Technology 2012 Clinic Summary Report. https://www.sartcorsonline.com/.

[6] Salim R., Riris S., Saab W., Abramov B., Khadum I., Serhal P. (2012). Adenomyosis reduces pregnancy rates in infertile women undergoing IVF. *Reproductive BioMedicine Online*.

[7] Thalluri V., Tremellen K. P. (2012). Ultrasound diagnosed adenomyosis has a negative impact on successful implantation following GnRH antagonist IVF treatment. *Human Reproduction*.

[8] Kuivasaari P., Hippeläinen M., Anttila M., Heinonen S. (2005). Effect of endometriosis on IVF/ICSI outcome: stage III/IV endometriosis worsens cumulative pregnancy and live-born rates. *Human Reproduction*.

[9] Opøien H. K., Fedorcsak P., Omland A. K. (2012). In vitro fertilization is a successful treatment in endometriosis-associated infertility. *Fertility and Sterility*.

[10] Harb H. M., Gallos I. D., Chu J., Harb M., Coomarasamy A. (2013). The effect of endometriosis on *in vitro* fertilisation outcome: a systematic review and meta-analysis. *BJOG*.

[11] Karita M., Yamashita Y., Hayashi A. (2011). Does advanced-stage endometriosis affect the gene expression of estrogen and progesterone receptors in granulosa cells?. *Fertility and Sterility*.

[12] Somigliana E., Infantino M., Benedetti F., Arnoldi M., Calanna G., Ragni G. (2006). The presence of ovarian endometriomas is associated with a reduced responsiveness to gonadotropins. *Fertility and Sterility*.

[13] Al-Azemi M., Bernai A. L., Steele J., Gramsbergen I., Barlow D., Kennedy S. (2000). Ovarian response to repeated controlled stimulation in in-vitro fertilization cycles in patients with ovarian endometriosis. *Human Reproduction*.

[14] Benaglia L., Bermejo A., Somigliana E. (2013). In vitro fertilization outcome in women with unoperated bilateral endometriomas. *Fertility and Sterility*.

[15] Suzuki T., Izumi S.-I., Matsubayashi H., Awaji H., Yoshikata K., Makino T. (2005). Impact of ovarian endometrioma on oocytes and pregnancy outcome in in vitro fertilization. *Fertility and Sterility*.

[16] Almog B., Shehata F., Sheizaf B., Tan S. L., Tulandi T. (2011). Effects of ovarian endometrioma on the number of oocytes retrieved for in vitro fertilization. *Fertility and Sterility*.

[17] Filippi F., Benaglia L., Paffoni A. (2014). Ovarian endometriomas and oocyte quality: insights from in vitro fertilization cycles. *Fertility and Sterility*.

[18] Olivennes F., Feldberg D., Liu H. C., Cohen J., Moy F., Rosenwaks Z. (1995). Endometriosis: a stage by stage analysis—the role of in vitro fertilization. *Fertility and Sterility*.

[19] Ballester M., Oppenheimer A., Mathieu D'Argent E. (2012). Deep infiltrating endometriosis is a determinant factor of cumulative pregnancy rate after intracytoplasmic sperm injection/in vitro fertilization cycles in patients with endometriomas. *Fertility and Sterility*.

[20] Ballester M., Oppenheimer A., D'Argent E. M. (2012). Nomogram to predict pregnancy rate after ICSI-IVF cycle in patients with endometriosis. *Human Reproduction*.

[21] Benaglia L., Somigliana E., Santi G., Scarduelli C., Ragni G., Fedele L. (2011). IVF and endometriosis-related symptom progression: insights from a prospective study. *Human Reproduction*.

[22] D'Hooghe T. M., Denys B., Spiessens C., Meuleman C., Debrock S. (2006). Is the endometriosis recurrence rate increased after ovarian hyperstimulation?. *Fertility and Sterility*.

[23] Surrey E. S., Silverberg K. M., Surrey M. W., Schoolcraft W. B. (2002). Effect of prolonged gonadotropin-releasing hormone agonist therapy on the outcome of in vitro fertilization-embryo transfer in patients with endometriosis. *Fertility and Sterility*.

[24] Remorgida V., Anserini P., Croce S., Costa M., Ferraiolo A., Capitanio G. L. (1990). Comparison of different ovarian stimulation protocols for gamete intrafallopian transfer in patients with minimal and mild endometriosis. *Fertility and Sterility*.

[25] Dicker D., Goldman G. A., Ashkenazi J., Feldberg D., Voliovitz I., Goldman J. A. (1990). The value of pre-treatment with gonadotrophin releasing hormone (GnRH) analogue in IVF-ET therapy of severe endometriosis. *Human Reproduction*.

[26] Nakamura K., Oosawa M., Kondou I. (1992). Menotropin stimulation after prolonged gonadotropin releasing hormone agonist pretreatment for in vitro fertilization in patients with endometriosis. *Journal of Assisted Reproduction and Genetics*.

[27] Marcus S. F., Edwards R. G. (1994). High rates of pregnancy after long-term down-regulation of women with severe endometriosis. *American Journal of Obstetrics and Gynecology*.

[28] Chedid S., Camus M., Smitz J., Van Steirteghern A. C., Devroey P. (1995). Comparison among different ovarian stimulation regimens for assisted procreation procedures in patients with endometriosis. *Human Reproduction*.

[29] Rickes D., Nickel I., Kropf S., Kleinstein J. (2002). Increased pregnancy rates after ultralong postoperative therapy with gonadotropin-releasing hormone analogs in patients with endometriosis. *Fertility and Sterility*.

[30] Sallam H. N., Garcia-Velasco J. A., Dias S., Arici A. (2006). Long-term pituitary down-regulation before in vitro fertilization (IVF) for women with endometriosis. *Cochrane Database of Systematic Reviews*.

[31] van der Houwen L. E. E., Mijatovic V., Leemhuis E. (2014). Efficacy and safety of IVF/ICSI in patients with severe endometriosis after long-term pituitary down-regulation. *Reproductive BioMedicine Online*.

[32] Ferrero S., Gillott D. J., Remorgida V., Anserini P., Ragni N., Grudzinskas J. G. (2009). GnRH analogue remarkably down-regulates inflammatory proteins in peritoneal fluid proteome of women with endometriosis. *Journal of Reproductive Medicine for the Obstetrician and Gynecologist*.

[33] Sharpe-Timms K. L., Keisler L. W., McIntush E. W., Keisler D. H. (1998). Tissue inhibitor of metalloproteinase-1 concentrations are attenuated in peritoneal fluid and sera of women with endometriosis and restored in sera by gonadotropin-releasing hormone agonist therapy. *Fertility and Sterility*.

[34] Bilotas M., Barañao R. I., Buquet R., Sueldo C., Tesone M., Meresman G. (2007). Effect of GnRH analogues on apoptosis and expression of Bcl-2, Bax, Fas and FasL proteins in endometrial epithelial cell cultures from patients with endometriosis and controls. *Human Reproduction*.

[35] Wang J., Zhou F., Dong M., Wu R., Qian Y. (2006). Prolonged gonadotropin-releasing hormone agonist therapy reduced expression of nitric oxide synthase in the endometrium of women with endometriosis and infertility. *Fertility and Sterility*.

[36] Lessey B. A. (2000). Medical management of endometriosis and infertility. *Fertility and Sterility*.

[37] Ruan H.-C., Zhu X.-M., Luo Q. (2006). Ovarian stimulation with GnRH agonist, but not GnRH antagonist, partially restores the expression of endometrial integrin *β*
_3_ and leukaemia-inhibitory factor and improves uterine receptivity in mice. *Human Reproduction*.

[38] Farrell R., Gray D., Gindlesperger V., Arredondo F., Liu J., de Mola J. R. L. (2003). Treatment of *α*V*β*3 integrin abnormalities increases pregnancy success rates after failed IVF cycles. *Fertility and Sterility*.

[39] Surrey E. S., Minjarez D. A., Schoolcraft W. B. (2007). The incidence of aberrant endometrial *α*v*β*
_3_ vitronectin expression in a high risk infertility population: could prolonged GnRH agonist therapy play a role?. *Journal of Assisted Reproduction and Genetics*.

[40] Surrey E. S., Lietz A. K., Gustofson R. L., Minjarez D. A., Schoolcraft W. B. (2010). Does endometrial integrin expression in endometriosis patients predict enhanced in vitro fertilization cycle outcomes after prolonged GnRH agonist therapy?. *Fertility and Sterility*.

[41] Raziel A., Schachter M., Strassburger D., Bern O., Ron-El R., Friedler S. (2007). Favorable influence of local injury to the endometrium in intracytoplasmic sperm injection patients with high-order implantation failure. *Fertility and Sterility*.

[42] Tei C., Maruyama T., Kuji N., Miyazaki T., Mikami M., Yoshimura Y. (2003). Reduced expression of *α*v*β*3 integrin in the endometrium of unexplained infertility patients with recurrent IVF-ET failures: improvement by danazol treatment. *Journal of Assisted Reproduction and Genetics*.

[43] Miller P. B., Parnell B. A., Bushnell G. (2012). Endometrial receptivity defects during IVF cycles with and without letrozole. *Human Reproduction*.

[44] de Ziegler D., Gayet V., Aubriot F. X. (2010). Use of oral contraceptives in women with endometriosis before assisted reproduction treatment improves outcomes. *Fertility and Sterility*.

[45] Franasiak J. M., Holoch K. J., Yuan L., Schammel D. P., Young S. L., Lessey B. A. (2014). Prospective assessment of midsecretory endometrial leukemia inhibitor factor expression versus *ανβ*
_3_ testing in women with unexplained infertility. *Fertility and Sterility*.

[46] Surrey E. S., Schoolcraft W. B. (2003). Does surgical management of endometriosis within 6 months of an in vitro fertilization-embryo transfer cycle improve outcome?. *Journal of Assisted Reproduction and Genetics*.

[47] Bedaiwy M. A., Falcone T., Katz E., Goldberg J. M., Assad R., Thornton J. (2008). Association between time from endometriosis surgery and outcome of *in vitro* fertilization cycles. *Journal of Reproductive Medicine for the Obstetrician and Gynecologist*.

[48] Opøien H. K., Fedorcsak P., Byholm T., Tanbo T. (2011). Complete surgical removal of minimal and mild endometriosis improves outcome of subsequent IVF/ICSI treatment. *Reproductive BioMedicine Online*.

[49] Barri P. N., Coroleu B., Tur R., Barri-Soldevila P. N., Rodríguez I. (2010). Endometriosis-associated infertility: surgery and IVF, a comprehensive therapeutic approach. *Reproductive BioMedicine Online*.

[50] Bianchi P. H. M., Pereira R. M. A., Zanatta A., Alegretti J. R., Motta E. L. A., Serafini P. C. (2009). Extensive excision of deep infiltrative endometriosis before *in vitro* fertilization significantly improves pregnancy rates. *Journal of Minimally Invasive Gynecology*.

[51] Douay-Hauser N., Yazbeck C., Walker F., Luton D., Madelenat P., Koskas M. (2011). Infertile women with deep and intraperitoneal endometriosis: comparison of fertility outcome according to the extent of surgery. *Journal of Minimally Invasive Gynecology*.

[52] Johnson N., van Voorst S., Sowter M. C., Strandell A., Mol B. W. J. (2010). Surgical treatment for tubal disease in women due to undergo in vitro fertilisation. *The Cochrane Database of Systematic Reviews*.

[53] Surrey E. S., Schoolcraft W. B. (2001). Laparoscopic management of hydrosalpinges before in vitro fertilization-embryo transfer: salpingectomy versus proximal tubal occlusion. *Fertility and Sterility*.

[54] Galen D. I., Khan N., Richter K. S. (2011). Essure multicenter off-label treatment for hydrosalpinx before *in vitro* fertilization. *Journal of Minimally Invasive Gynecology*.

[55] Brosens I., Gordts S., Puttemans P., Benagiano G. (2014). Pathophysiology proposed as the basis for modern management of the ovarian endometrioma. *Reproductive BioMedicine Online*.

[56] Sanchez A. M., Viganò P., Somigliana E., Panina-Bordigno P., Vercellini P., Candiani M. (2014). The distinguishing cellular and molecular features of the endometriotic ovarian cyst: frompathophysiology to the potential endometrioma-mediated damage to the ovary. *Human Reproduction Update*.

[57] Khamsi F., Yavas Y., Lacanna I. C., Roberge S., Endman M., Wong J. C. (2001). GYNECOLOGY: exposure of human oocytes to endometrioma fluid does not alter fertilization or early embryo development. *Journal of Assisted Reproduction and Genetics*.

[58] Tsoumpou I., Kyrgiou M., Gelbaya T. A., Nardo L. G. (2009). The effect of surgical treatment for endometrioma on in vitro fertilization outcomes: a systematic review and meta-analysis. *Fertility and Sterility*.

[59] Benschop L., Farquhar C., van der Poel N., Heineman M. J. (2010). Interventions for women with endometrioma prior to assisted reproductive technology. *The Cochrane Database of Systematic Reviews*.

[60] Elter K., Oral E. (2014). Surgical treatment before assisted reproductive technologies. *Seminars in Reproductive Medicine*.

[61] Garcia-Velasco J. A., Somigliana E. (2009). Management of endometriomas in women requiring IVF: to touch or not to touch. *Human Reproduction*.

[62] Tang Y., Chen S.-L., Chen X. (2013). Ovarian damage after laparoscopic endometrioma excision might be related to the size of cyst. *Fertility and Sterility*.

[63] Somigliana E., Ragni G., Benedetti F., Borroni R., Vegetti W., Crosignani P. G. (2003). Does laparoscopic excision of endometriotic ovarian cysts significantly affect ovarian reserve? Insights from IVF cycles. *Human Reproduction*.

[64] Benaglia L., Somigliana E., Vighi V., Ragni G., Vercellini P., Fedele L. (2010). Rate of severe ovarian damage following surgery for endometriomas. *Human Reproduction*.

[65] Somigliana E., Berlanda N., Benaglia L., Viganò P., Vercellini P., Fedele L. (2012). Surgical excision of endometriomas and ovarian reserve: a systematic review on serum antimüllerian hormone level modifications. *Fertility and Sterility*.

[66] Muzii L., di Tucci C., di Feliciantonio M. (2014). The effect of surgery for endometrioma on ovarian reserve evaluated by antral follicle count: a systematic review and meta-analysis. *Human Reproduction*.

